# Mentally active versus passive sedentary behaviors and burnout among nurses of China: a cross-sectional study

**DOI:** 10.3389/fpubh.2026.1803165

**Published:** 2026-04-21

**Authors:** Jiangqin He, Peige Song, Cao Zhang, Lili Yang

**Affiliations:** 1 Department of Nursing, the Fourth Affiliated Hospital of School of Medicine, and International School of Medicine, International Institutes of Medicine, Zhejiang University, Yiwu, China; 2 School of Public Health, Zhejiang University School of Medicine, Hangzhou, China; 3 Department of Anesthesia, the Fourth Affiliated Hospital of School of Medicine, and International School of Medicine, International Institutes of Medicine, Zhejiang University, Yiwu, China

**Keywords:** burnout, cross-sectional study, mental engagement, nurses, sedentary behaviors

## Abstract

**Purpose:**

The aim of this study was to explore the differential associations of mentally active versus passive sedentary behavior with burnout among nurses.

**Methods:**

A cross-sectional study was conducted among 1,132 nurses in Zhejiang, China, daily SBs were self-reported with The Chinese Adult Sedentary Behavior Questionnaire and burnout was measured by Maslach Burnout Inventory-Human Services Survey.

**Results:**

Nurses who accumulated more than 4 h/day of mentally active SBs (working, reading, hobbies, transportation, and chatting) had 47% lower burnout odds than those who reported less than1 h/day (OR = 0.53; 95% CI 0.33–0.86); this inverse association remained after excluding transport-related SB. Mentally passive SB showed no significant association.

**Conclusion:**

Engaging in mentally active sedentary behavior was associated with lower odds of burnout among nurses.

## 1 Introduction

Burnout is a work-related syndrome characterized by emotional exhaustion, depersonalization, and a sense of reduced accomplishment (1) and represents a major concern in nurses (2). Population-based surveys indicate prevalence estimates of 10% in Netherlands and 78% in Greece (3), and as high as 52% in Asia (4). Aiken et al. (3) demonstrated that each additional patient per nurse is associated with 23% increased odds of burnout. Accumulating evidence further links nurse burnout to adverse patient outcomes, including heightened mortality, failure-to-rescue events, and prolonged length of stay (5–7). These findings emphasize the crucial need to address nurse burnout as a major barrier to the quality of care provided; burnout may be modified by potentially modifiable daily behaviors—among which sedentary time remains largely overlooked.

Sedentary behaviors (SBs) were defined as “any waking behavior characterized by an energy expenditure ≤1.5 METs (metabolic equivalent units) while in a sitting or reclining posture.” (8) When adult are not physically active, they tend to spend a great deal of time in SBs (9). Hallgren et al. (10) classified SB according to the level of mental engagement involved, partitioning it into mentally active SB (e.g., reading, writing, computer-mediated tasks) and mentally passive SB (e.g., television viewing, music listening). Studies suggest that mentally active SB may confer psychological benefit, whereas passive SB is consistently associated with deleterious mental-health outcomes (11). Thus, mentally active SB could act as a recovery strategy, whereas mentally passive SB may compound psychosocial strain; however, this dichotomy has not been tested in nursing populations.

Indeed, the association between SBs and burnout is inconsistent across occupations. Morgan et al. (12) observed that medical students accumulating greater daily sedentary time reported unexpectedly lower burnout scores, implying that sitting may operate as a recovery mechanism rather than a uniformly hazardous exposure. That analysis, however, relied on a unidimensional metric of sitting time, leaving the qualitative dimensions of sedentary behavior unexamined. Verhavert et al. (13) disaggregated sedentary time into work-, transport-, and leisure-domain contexts and demonstrated that, among teachers, prolonged work-related sitting predicted higher burnout risk, whereas sitting accrued during commuting or leisure bore no significant association. This domain-based stratification obscures within-context heterogeneity in mental demand. As a teacher, leisure-period television viewing and lesson planning both occur during discretionary time but impose markedly different cognitive loads, thereby potentially masking divergent effects on burnout. Among nurses—an occupational group characterized by high burnout prevalence and sedentary profiles comprising 50%–60% of waking hours (14)—there remains a clear imperative to reclassify SB along the dimension of mental engagement. Distinguishing mentally active from passive SB may elucidate modality-specific contributions to burnout risk.

Our objective was to disentangle the differential associations of mentally active versus passive SBs with burnout among nurses, thereby establishing an evidence base for precision workplace interventions that target sedentary modality rather than volume alone.

## 2 Methods

### 2.1 Study design and participants

This cross-sectional survey was conducted in Zhejiang, China, from March 2023 to April 2023 using a multistage, stratified-sampling method. In stage 1, we used random number method to select seven cities out of the 11 cities in Zhejiang. In stage 2, we randomly selected one to two tertiary-level hospitals from each region. In stage 3, we recruited 100 registered nurses in each hospital to complete the questionnaire. Participants were recruited through WeChat and newsletters, with a voluntary requirement that they be over 18 years old and work in medical institutions within Zhejiang Province. The online questionnaire was distributed to 1,300 eligible individuals. Only participants who completed the survey were included. The confidentiality and anonymity of all nurses in this study were ensured.

### 2.2 Research tools

#### 2.2.1 Assessment of sedentary behaviors

The Chinese Adult Sedentary Behavior Questionnaire was used to assess sedentary time and type. This questionnaire was designed by an interdisciplinary group of Sports Health and Health Promotion scientists. The test–retest reliability intra-class-coefficients (ICC) of the questionnaire was 0.82, and the Spearman correlation coefficient was 0.71 (15). Participants reported the average time each day spent sitting and engaged in 10 activities over the previous 7 days, including work, browsing websites, mealtime, napping, reading (books, newspapers and magazines), hobbies times, transportation, chatting, watching TV, other sitting times. The average daily hours spent on each SBs were calculated as (days of specific SB in the past 7 days) * (the average daily time on a specific SB during those days)/7. Participants who reported a total sedentary time exceeding 24 h per day or 0 h of mealtime sedentary behavior were excluded. This a priori decision was based on the rationale that such extreme values were more likely to represent measurement error or a misunderstanding of the questionnaire items.

Based on the literature review (16–18),we categorized watching TV, browsing websites, having meals, and napping as mentally passive sedentary behaviors, and working, reading, hobbies, transportation, and chatting as mentally active sedentary behaviors. The time spent on mentally passive SBs was divided into four categories (0 to 1, 1 to 2, 2 to 3, and>3 per day), while the time spent on mentally active SBs was divided into five categories (0 to1, 1 to 2, 2 to 3, 3 to 4, and >4 per day), as previously found (10, 19, 20).

#### 2.2.2 Assessment of burnout

Burnout was evaluated using the Maslach Burnout Inventory - Human Services Survey (MBI-HSS), which comprises 22 items rated on a scale of 0–6 points for each item (0 = never to 6 = every day) (21). The Chinese version of the inventory had a reliability of 0.9, 0.88 and 0.68 for EE, PA and DP, respectively (22). This inventory is widely used in healthcare personnel, and has a high level of internal consistency. The Cronbach’s alpha coefficient for each dimension is 0.90 for Emotional Exhaustion (EE), 0.79 for Depersonalization (DP), and 0.71 for Personal Accomplishment (PA) (23, 24).

The MBI-HSS measures burnout syndrome across three dimensions: EE with 9 items, DP with 5 items, and PA with 8 items. According to the MBI-HSS questionnaire manual (21), the scores above 26 for EE, above 9 for DP, and below 34 for PA indicate the presence of burnout in the respective dimensions. Scores for EE and DP are directly proportional to the intensity of the burnout syndrome, while the PA score is inversely proportional (25). Healthcare professionals who experience high levels of burnout can be identified by the pattern of simultaneously scoring high on the dimensions of EE and DP, while scoring low on the dimension of PA (26). A characteristic pattern for severe burnout is the co-occurrence of high scores on both EE and DP alongside a low score on PA. Therefore, for the purpose of this study, an operational definition of “burnout” was applied: participants were classified into the burnout group only if they simultaneously met the high-threshold criteria for all three dimensions (i.e., EE > 26, DP > 9, and PA < 34). All other participants were categorized into the non-burnout group.

#### 2.2.3 Assessments of other covariates

A self-administered questionnaire was designed to collect sociodemographic information. Including various factors such as age (≤30, >30–≤40, >40), gender, education background (vocational or below, undergraduate or higher), monthly income, smoking habits (yes or no), alcohol consumption (yes or no) in the previous year (2023), and marital status (never married, married, widowed or divorced), number of children (0, 1, 2, or ≥3), job title (junior, senior, deputy chief, or higher), years of work experience, frequency of night shifts (≤4, >4–<10, ≥10 per month), duration of sleep (≤7, >7–<9, ≥9 h per day), self-perceived health status and body mass index (BMI). Self-perceived health status was evaluated based on the participant’s own perception and a comparison with peers of the same age. It was categorized into four levels: much better, better, same, worse, or much worse. The BMI were determined based on the criteria established by the Working Group on Obesity in China (WGOC). These categories include underweight for BMI ≤ 18.5, normal weight for 18.5 < BMI ≤ 24, overweight for 24 < BMI ≤ 28, and obese for BMI > 28 (27).

### 2.3 Statistical analysis

All statistical analyses were performed using the R software (version 4.3.2), and the significance level was established at a p value less than 0.05. Descriptive statistics were employed to present the data, detailing frequencies (*N*) and percentages (%) for categorical variables, and the mean ± standard deviation for continuous variables. To compare the burnout and non-burnout groups, we conducted chi-squared tests for categorical variables and t-tests for continuous variables. Multivariable logistic regression analysis was employed to estimate odds ratios (ORs) with 95% confidence intervals (CIs) to examine the association between mentally active and mentally passive SBs and burnout.

Three logistic regression models were formulated, each with a reference group of ≥0-≤1 h of SB. Mentally active SBs were stratified into five categories (≥0-≤1, >1-≤2, >2-≤3, >3-≤4, and >4 h per day), while mentally passive SB was categorized into four (≥0-≤1, >1-≤2, >2-≤3 and >3 h per day). Model 1 was unadjusted, Model 2 included adjustments for age, sex, body mass index (BMI), education, monthly income, marital status, number of children, and habits such as smoking and drinking, along with health status. Model 3 incorporated additional covariates: job title, frequency of night shifts, sleep duration, and years of professional experience.

To assess the influence of specific mentally active SB domains on burnout, we performed multivariable logistic regression analyses for each domain (e.g., work, reading). For these subgroup analyses, the reference category within each domain was set as individuals reporting 0 h/day of that specific activity. All models were adjusted for the covariates previously described.

## 3 Results

### 3.1 Sociodemographic characteristic of the participants

**Figure 1:**
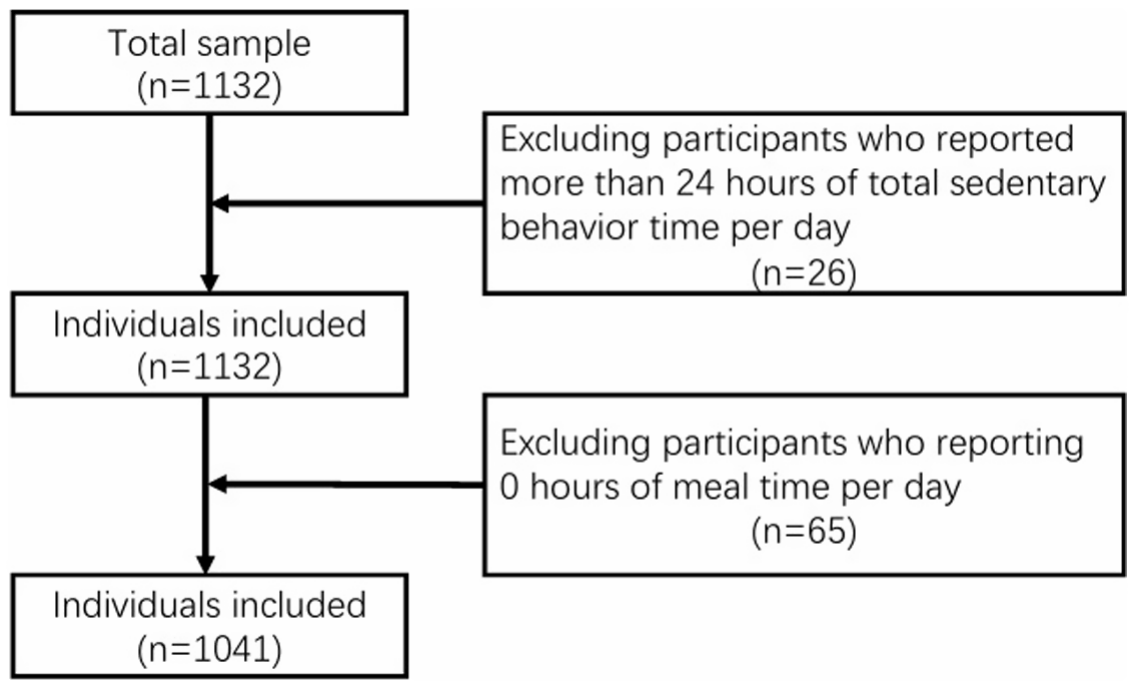
Flow chart.

A total of 1,132 nurses completed the online questionnaire, yielding a valid response rate of 87.1%. After excluding participants who reported more than 24 h of total sedentary behavior time per day (*n* = 26) and those reporting 0 h of mealtime per day (*n* = 65), 1,041 questionnaires were considered valid (rate of eligible questionnaires: 92%) (Figure 1).

Table 1 shows the sociodemographic characteristics of the study participants. A total of 1,041 Chinese nurses, 204 (19.60%) met the criteria for burnout. Compared to the non-burnout group, nurses with burnout had significantly shorter sleep duration, poorer self- perceived health status, and less engagement in mentally active SBs (*p* < 0.05). No significant intergroup differences were observed for age, BMI, job title, working years, night shift frequency, or engagement in mentally passive SBs (*p* > 0.05).

**Table 1:** Characteristics of the participants.

	Not burnout (*n* = 837)	Burnout (*n* = 204)	*p*
Age (%)			0.238
≤30	448 (53.5)	119 (58.3)	
>30–≤40	297 (35.5)	70 (34.3)	
>40	92 (11.0)	15 (7.4)	
Sex (%)			0.423
Men	28 (3.3)	4 (2.0)	
Women	809 (96.7)	200 (98.0)	
BMI (kg/m^2^) (%)			0.569
<18.5	96 (11.5)	29 (14.2)	
≥18.5–<24.0	530 (63.3)	129 (63.2)	
≥24.0–<28.0	149 (17.8)	35 (17.2)	
≥28.0	62 (7.4)	11 (5.4)	
Education (%)			0.647
Vocational or below	194(23.2)	51(25.0)	
Undergraduate or above	643(76.8)	153(75.0)	
Monthly income (%)			0.487
≤4,000	90 (10.8)	29 (14.2)	
>4,000–≤6,000	312 (37.3)	80 (39.2)	
>6,000–≤8,000	263 (31.4)	56 (27.5)	
>8,000–≤10,000	108 (12.9)	27 (13.2)	
>10,000	64 (7.6)	12 (5.9)	
Marital status (%)			0.607
Never married	323 (38.6)	85 (41.7)	
Married	498 (59.5)	114 (55.9)	
Widowed or divorced	16 (1.9)	5 (2.5)	
Number of children (%)			0.272
0	368 (44.0)	101 (49.5)	
1	264 (31.5)	54 (26.5)	
2	202 (24.1)	47 (23.0)	
≥3	3 (0.4)	2 (1.0)	
Job title (%)			0.57
Junior nurse	492 (58.8)	128 (62.7)	
Senior nurse	296 (35.4)	66 (32.4)	
Deputy chief nurse or above	49 (5.9)	10 (4.9)	
Working years (mean (SD))	10.05 (7.48)	9.59 (6.85)	0.427
Night shifts (time/month) (%)			0.519
≤4	402 (48.0)	91 (44.6)	
>4–<10	330 (39.4)	82 (40.2)	
≥10	105 (12.5)	31 (5.2)	
Sleep duration (hour/day) (%)			<0.001*
≤7	470 (56.2)	163 (79.9)	
>7–<9	359 (42.9)	41 (20.1)	
≥9	8 (1.0)	0 (0.0)	
Smoke (%)			0.386
No	826 (98.7)	199 (97.5)	
Yes	11 (1.3)	5 (2.5)	
Drink (%)			0.607
No	790 (94.4)	190 (93.1)	
Yes	27 (5.6)	14 (6.9)	
Healthy compared to others (%)			<0.001*
Much better	20 (2.4)	3 (1.5)	
Better	105 (12.5)	12 (5.9)	
Same	471 (56.3)	72 (35.3)	
Worse	206 (24.6)	86 (42.2)	
Much worse	35 (4.2)	31 (15.2)	
Mentally active SBs (h/d)			0.016*
≥0-≤1	98 (11.7)	41 (20.1)	
>1-≤2	128 (15.3)	30 (14.7)	
>2-≤3	95 (11.4)	27 (13.2)	
>3-≤4	66 (7.9)	16 (7.8)	
>4	450 (53.8)	90 (44.1)	
Mentally passive SBs (h/d)			0.078
≥0-≤1	48 (5.7)	19 (9.3)	
>1-≤2	97 (11.6)	30 (14.7)	
>2-≤3	111 (13.3)	31 (15.2)	
>3	581 (69.4)	124 (60.8)	

*p < 0.05.

### 3.2 Association between different domains of SBs and burnout

Table 2 shows the association between different domains of SBs and burnout. Participants with burnout spent less time on mentally active SBs, while no statistically significant difference was found for mentally passive SBs (Supplementary Figure 1; Supplementary Table 1). In the unadjusted model, participants who spent four or more hours on mentally active SBs had lower odds of burnout compared to those with less than 1 h (OR = 0.48, 95% CI = 0.31–0.74), those who spent 1-2 h on mentally active SBs had an OR of 0.56 (95% CI = 0.32–0.96). After full adjustment (Model 3), the association for >4 h of mentally active SB remained significant, with 47% lower odds of burnout (OR = 0.53, 95% CI = 0.33–0.86). No statistically significant associations were found for the other SB categories, except for participants who spent three or more hours on mentally passive SBs. While there was a decreased odds of burnout in mentally passive SBs (OR = 0.54, 95% CI = 0.31–0.97) compared to those who spent 1 h or less per day in mentally passive SBs in model 1, this was not found in models 2 and 3.

**Table 2:** Association between SBs and burnout.

	Model 1		Model 2		Model 3	
	OR (95% CI)	*p*-value	OR (95% CI)	*p*-value	OR (95% CI)	*p*-value
Mentally active SBs						
≥0-≤1	Ref		Ref		Ref	
>1-≤2	0.56 (0.32, 0.96)	0.035^*^	0.59 (0.33, 1.05)	0.074	0.62 (0.35, 1.11)	0.110
>2-≤3	0.68 (0.38, 1.19)	0.177	0.78 (0.42, 1.41)	0.412	0.79 (0.42, 1.46)	0.451
>3-≤4	0.58 (0.29, 1.1)	0.103	0.65 (0.32, 1.3)	0.231	0.71 (0.34, 1.44)	0.350
>4	0.48 (0.31, 0.74)	<0.001^*^	0.52 (0.33, 0.83)	0.006^*^	0.53 (0.33, 0.86)	0.009^*^
Mentally passive SBs						
≥0-≤1	Ref		Ref		Ref	
>1-≤2	0.78 (0.4, 1.54)	0.471	0.83 (0.4, 1.72)	0.608	0.85 (0.41, 1.76)	0.651
>2-≤3	0.71 (0.37, 1.39)	0.303	0.79 (0.39, 1.61)	0.505	0.84 (0.41, 1.74)	0.639
>3	0.54 (0.31, 0.97)	0.032^*^	0.59 (0.32, 1.11)	0.094	0.65 (0.35, 1.22)	0.167

Model 1 not adjusted.

Model 2 adjusted for age, sex, BMI, education, monthly income, marital, children, smoke, drink, health status.

Model 3: adjusted for all variables in Model 2 plus title, night shifts, sleep duration, and working years.

*p < 0.05.

### 3.3 Subgroup analysis of mentally active SBs and burnout

Table 3 presents a subgroup analysis of mentally active SBs and burnout. Significant associations were found for mentally active SBs in all subgroups except transportation (Supplementary Figure 2). After adjusting for covariates, participants who engaged in working SBs for 1 hour or more had a decreased odds of burnout (OR = 0.51, 95% CI = 0.31–0.97). Model 1 showed that the ORs for reading, hobbies, and chatting were 0.5 (95% CI = 0.31–0.97), 0.52 (95% CI = 0.35–0.76), and 0.41 (95% CI = 0.29–0.59), respectively, compared to participants who did not engage in these SBs. After the final adjustment (Model 3), the odds of burnout were reduced by 0.57 (95% CI = 0.4–0.82), 0.66 (95% CI = 0.43–0.99), and 0.48 (95% CI = 0.33–0.71) for participants who spent less than 1 hour per day reading, participating in hobbies, and chatting with others, respectively, compared to those who did not engage in these activities.

**Table 3:** Association between mentally active SBs and burnout.

	Model 1		Model 2		Model 3	
	OR (95% CI)	*p*-value	OR (95% CI)	*p*-value	OR (95% CI)	*p*-value
Working						
0	Ref		Ref		Ref	
>0-≤1	0.6 (0.38, 0.95)	0.030^*^	0.69 (0.42, 1.13)	0.139	0.72 (0.43, 1.18)	0.190
>1	0.48 (0.32, 0.72)	<0.001^*^	0.52 (0.34, 0.8)	0.003^*^	0.51 (0.33, 0.8)	0.003^*^
Reading						
0	Ref		Ref		Ref	
>0-≤1	0.5 (0.35, 0.7)	<0.001^*^	0.58 (0.4, 0.82)	0.003^*^	0.57 (0.4, 0.82)	0.003^*^
>1	0.58 (0.26, 1.17)	0.153	0.58 (0.25, 1.22)	0.176	0.59 (0.25, 1.25)	0.190
Hobbies						
0	Ref		Ref		Ref	
>0-≤1	0.52 (0.35, 0.76)	<0.001^*^	0.62 (0.41, 0.93)	0.024^*^	0.66 (0.43, 0.99)	0.050^*^
>1	1.1 (0.55, 2.04)	0.783	1.46 (0.71, 2.82)	0.280	1.5 (0.72, 2.96)	0.258
Transportation						
0	Ref		Ref		Ref	
>0-≤1	0.94 (0.66, 1.37)	0.755	0.95 (0.65, 1.41)	0.802	1 (0.68, 1.5)	0.987
>1	1.08 (0.65, 1.78)	0.770	1.02 (0.59, 1.73)	0.949	1.09 (0.63, 1.88)	0.748
Chatting						
0	Ref		Ref		Ref	
>0-≤1	0.41 (0.29, 0.59)	<0.001^*^	0.42 (0.29, 0.62)	<0.001^*^	0.48 (0.33, 0.71)	<0.001^*^
>1	0.55 (0.31, 0.95)	0.035^*^	0.6 (0.33, 1.07)	0.087	0.73 (0.39, 1.32)	0.299

Model 1 not adjusted.

Model 2 adjusted for age sex, BMI, education, monthly income, marital, children, smoke, drink, health status.

Model 3: adjusted for all variables in Model 2 plus title, night shifts, sleep duration, and working years.

*p < 0.05.

## 4 Discussion

This is the first investigation to dissect the relationship between domain-specific SBs and burnout among nurses. Mentally active SBs were independently associated with lower odds of burnout, whereas mentally passive SBs showed no significant association. These data corroborate previous reports that distinct SB domains exert divergent effects on health and well-being (10, 18). Our findings underscore the importance of qualitatively reclassifying SB when designing burnout-prevention strategies.

The observed burnout prevalence of 19.6% is lower than China (27.7%) (28) and Singapore (24%) (29). Although cross-national differences in socio-economic status and living conditions have been invoked to explain such heterogeneity (28), income was not predictive of burnout in the present study (*p* > 0.05). This null finding may reflect a ceiling effect, as participating nurses earned substantially more than their counterparts in other studies. As found in the literature (30) that a higher economic status is associated with better health conditions. In our study, we found that the nurses who rated their self-perceived health status highly had lower burnout rates (*p* < 0.05). Thus, we infer that self-rated health remained inversely associated with burnout regardless of income, underscoring the primacy of perceived physical and mental well-being.

Consistent with prior work linking prolonged SB to heightened burnout (13, 31), we found that only mentally active SB exceeding 4 h/day was associated with lower odds of burnout, mentally passive SB had no significant association with burnout. The result as posited by Morgan et al. (12) and suggest that sedentary time may be a recovery mechanism. When nurses engage in mentally active sedentary behaviors(hobby cultivation, supportive conversation with family members), they accrue greater psychosocial dividends—stress release, emotional support, self-affirmation—that can attenuate, or even offset, work-induced fatigue, thereby mitigating burnout risk. In our study, we found that—excluding transportation-related sitting—nurses who engaged in less than 1 h/day of mentally active SBs such as reading, hobbies, and chatting or more than 1 h/day working-related SBs had a reduced odds of burnout compared with those who did not participate in these activities. Conversely, mentally passive screen-based exposure, such as television viewing or undirected web browsing conferred no benefit. Therefore, interventions should educate nurses to participate in more mentally active SBs while curtailing television viewing and aimless web-surfing.

Moreover, our study found a significant relationship between sleep duration and burnout (*p* < 0.001), which is consistent with previous research (32). Chin et al.’s (33) research further suggests a dose-dependent relationship, indicating that nurses who sleep less than 6 h per workday evidenced a threefold higher odd of burnout (AOR = 3.4, 95% CI = 2.0–6.0) relative to those sleep more than 7 h. In addition, it is worth to paying attention to sleep quality. A study of a large healthcare cluster in Singapore (*N* = 4,777) investigated the relationship between sleep quality and burnout using the Pittsburgh Sleep Quality Index (34), revealed a strong correlation between poor sleep quality and all three burnout dimensions (EE: *F* = 90.65; DP: *F* = 49.46; PA: *F* = 12.29; all *p* < 0.0001). Therefore, optimizing both sleep duration and quality should be prioritized as a low-resistance burnout countermeasure.

Our findings highlight that not all domains of SBs are associated with burnout. In the nursing profession, engagement in mentally active SB was associated with lower odds of burnout. Further interventions should focus on reducing mentally passive SBs, such as TV viewing or aimlessly browsing the internet, while promoting an increase in mentally active SBs. Off-duty nurses should be encouraged to engage in academic exchange, continuing education, and hobby development; healthcare institutions must provide the infrastructural and temporal platforms required for such value-affirming activities.

While our study provides valuable insights, several limitations should be acknowledged. First, the cross-sectional design precludes causal inference, and reverse causality remains possible (e.g., nurses with lower burnout may engage in more mentally active sedentary behavior). Second, although multiple covariates were adjusted for, residual confounding from unmeasured or imprecisely measured factors cannot be excluded. Third, sedentary behavior was assessed using self-reported categorical measures, which may be subject to recall and social desirability bias. In addition, classifying activities as “mentally active” or “passive” may involve some misclassification, potentially attenuating the observed associations toward the null. Fourth, despite voluntary participation, selection and non-response bias may limit the representativeness of the sample. Finally, as all participants were recruited from tertiary hospitals within a single province, the findings may be influenced by unmeasured hospital-level factors. Future multicenter studies using objective measures across diverse healthcare settings are warranted to confirm and extend these findings.

## 5 Conclusion

We found that 19.6% of nurses reported burnout, which was significantly associated with mentally active SBs. Nurses engaging in more than 4 h/day of mentally active sedentary behavior had 47% lower odds of burnout, and this inverse association persisted after exclusion of transport-related SB. Interventions that extend mentally active SB, optimize sleep quantity and quality, and foster socially interactive pursuits should be prioritized to mitigate burnout in nursing staff.

## Data Availability

The original contributions presented in the study are included in the article/Supplementary material, further inquiries can be directed to the corresponding author.

## References

[1] Maslach C Schaufeli WB Leiter MP. Job burnout. Annu Rev Psychol. (2001) 52:397–422. doi: 10.1146/annurev.psych.52.1.397, 11148311

[2] Aiken LH Clarke SP Sloane DM Sochalski J Silber JH. Hospital nurse staffing and patient mortality, nurse burnout, and job dissatisfaction. JAMA. (2002) 288:1987–93. doi: 10.1001/jama.288.16.1987, 12387650

[3] Aiken LH Sermeus W den Van Heede K Sloane DM Busse R McKee M . Patient safety, satisfaction, and quality of hospital care: cross sectional surveys of nurses and patients in 12 countries in Europe and the United States. BMJ. (2012) 344:e1717. doi: 10.1136/bmj.e1717, 22434089 PMC3308724

[4] See KC Zhao MY Nakataki E Chittawatanarat K Fang WF Faruq MO . Professional burnout among physicians and nurses in Asian intensive care units: a multinational survey. Intensive Care Med. (2018) 44:2079–90. doi: 10.1007/s00134-018-5432-1, 30446797

[5] Schlak AE Aiken LH Chittams J Poghosyan L McHugh M. Leveraging the work environment to minimize the negative impact of nurse burnout on patient outcomes. Int J Environ Res Public Health. (2021) 18:610. doi: 10.3390/ijerph18020610, 33445764 PMC7828279

[6] Brooks Carthon JM Hatfield L Brom H Houton M Kelly-Hellyer E Schlak A . System-level improvements in work environments lead to lower nurse burnout and higher patient satisfaction. J Nurs Care Qual. (2021) 36:7–13. doi: 10.1097/NCQ.0000000000000475, 32102025 PMC7483185

[7] Poghosyan L Clarke SP Finlayson M Aiken LH. Nurse burnout and quality of care: cross-national investigation in six countries. Res Nurs Health. (2010) 33:288–98. doi: 10.1002/nur.20383, 20645421 PMC2908908

[8] Tremblay MS Aubert S Barnes JD Saunders TJ Carson V Latimer-Cheung AE . Sedentary behavior research network (SBRN) - terminology consensus project process and outcome. Int J Behav Nutr Phys Act. (2017) 14:75. doi: 10.1186/s12966-017-0525-8, 28599680 PMC5466781

[9] Sakakibara K Miyanaka D Tokita M Kawada M Mori N Hamsyah F . Association of work-related sedentary behavior with mental health and work engagement among Japanese White- and blue-collar workers. J Occup Environ Med. (2023) 65:e695–702. doi: 10.1097/JOM.0000000000002952, 37621026 PMC10662573

[10] Hallgren M Owen N Stubbs B Zeebari Z Vancampfort D Schuch F . Passive and mentally-active sedentary behaviors and incident major depressive disorder: a 13-year cohort study. J Affect Disord. (2018) 241:579–85. doi: 10.1016/j.jad.2018.08.020, 30170310

[11] Hallgren M Nguyen TT Owen N Stubbs B Vancampfort D Lundin A . Cross-sectional and prospective relationships of passive and mentally active sedentary behaviors and physical activity with depression. Br J Psychiatry. (2020) 217:413–9. doi: 10.1192/bjp.2019.60, 30895922

[12] Morgan TL McFadden T Fortier MS Sweet SN Tomasone JR. Do physical activity intensity and sedentary behaviour relate to burnout among medical students? Insight from two Canadian medical schools. Can Med Educ J. (2024) 15:54–63. doi: 10.36834/cmej.79169, 39588026 PMC11586019

[13] Verhavert Y Deliens T Van Cauwenberg J Van Hoof E Matthys C de Vries J . Associations of lifestyle with burnout risk and recovery need in Flemish secondary schoolteachers: a cross-sectional study. Sci Rep. (2024) 14:3268. doi: 10.1038/s41598-024-53044-w, 38332138 PMC10853556

[14] Prince SA Reid RD Bernick J Clarke AE Reed JL. Single versus multi-item self-assessment of sedentary behaviour: a comparison with objectively measured sedentary time in nurses. J Sci Med Sport. (2018) 21:925–9. doi: 10.1016/j.jsams.2018.01.018, 29500119

[15] Tian T Wen GB. Development and evaluation on reliability and validity of adult sedentary behavior questionaire in China. Chin J Health Educ. (2019) 35:525–9. doi: 10.16168/j.cnki.issn.1002-9982.2019.06.010

[16] Kikuchi H Inoue S Sugiyama T Owen N Oka K Nakaya T . Distinct associations of different sedentary behaviors with health-related attributes among older adults. Prev Med. (2014) 67:335–9. doi: 10.1016/j.ypmed.2014.08.011, 25117527

[17] Qi M Gao Y Zhao X Jones C Moyle W Shen S . Development and validity of a mentally-passive and mentally-active sedentary time questionnaire in nursing college students. Front Public Health. (2023) 11:1180853. doi: 10.3389/fpubh.2023.1180853, 37794895 PMC10546406

[18] Hallgren M Dunstan DW Owen N. Passive versus mentally active sedentary behaviors and depression. Exerc Sport Sci Rev. (2020) 48:20–7. doi: 10.1249/JES.0000000000000211, 31663866

[19] Werneck AO Owen N Araujo RHO Silva DR Hallgren M. Mentally-passive sedentary behavior and incident depression: mediation by inflammatory markers. J Affect Disord. (2023) 339:847–53. doi: 10.1016/j.jad.2023.07.053, 37467803

[20] Werneck AO Hoare E Stubbs B van Sluijs EMF Corder K. Associations between mentally-passive and mentally-active sedentary behaviors during adolescence and psychological distress during adulthood. Prev Med. (2021) 145:106436. doi: 10.1016/j.ypmed.2021.106436, 33485997 PMC7612670

[21] Maslach C Jackson SE. The measurement of experienced burnout. J Organ Behav. (1981) 2:99–113. doi: 10.1002/job.4030020205

[22] Lee HF Chien TW Yen M. Examining factor structure of Maslach burnout inventory among nurses in Taiwan. J Nurs Manag. (2013) 21:648–56. doi: 10.1111/j.1365-2834.2012.01427.x, 23410056

[23] Bassam S Mohsen H Barakat Z Abou-Abbas L. Psychometric properties of the Arabic version of the Maslach burnout inventory-human services survey (MBI-HSS) among Lebanese dentists. BMC Oral Health. (2023) 23:451. doi: 10.1186/s12903-023-03169-7, 37407968 PMC10324122

[24] Forne C Yuguero O. Factor structure of the Maslach burnout inventory human services survey in Spanish urgency healthcare personnel: a cross-sectional study. BMC Med Educ. (2022) 22:615. doi: 10.1186/s12909-022-03666-3, 35962362 PMC9373484

[25] Trigo TR de Freitas CCS Wang YP Ribeiro FG de Lucia MCS Siqueira JO . The influence of depression on the psychometric properties of the Maslach burnout inventory-human services survey: a cross-sectional study with nursing assistants. Front Psych. (2018) 9:695. doi: 10.3389/fpsyt.2018.00695, 30618870 PMC6305309

[26] Wang J Zhang L Jiang F Liu Y Wang M Wu Y . Gender differences in burnout among endocrinologists in China. Front Psychol. (2022) 13:845188. doi: 10.3389/fpsyg.2022.845188, 35300158 PMC8921076

[27] Ji CY Chen TJ Working Group on Obesity in C. Empirical changes in the prevalence of overweight and obesity among Chinese students from 1985 to 2010 and corresponding preventive strategies. Biomed Environ Sci. (2013) 26:1–12. doi: 10.3967/0895-3988.2013.01.001, 23294610

[28] Wei L Jing Z Fuye L Jiwen L. A survey on nurses’ job burnout in a 3A-level comprehensive hospitals. J Xinjiang Med Univ. (2014) 37:802–5.

[29] Teo I Chay J Cheung YB Sung SC Tewani KG Yeo LF . Healthcare worker stress, anxiety and burnout during the COVID-19 pandemic in Singapore: a 6-month multi-centre prospective study. PLoS One. (2021) 16:e0258866. doi: 10.1371/journal.pone.0258866, 34679110 PMC8535445

[30] Lange S Vollmer S. The effect of economic development on population health: a review of the empirical evidence. Br Med Bull. (2017) 121:47–60. doi: 10.1093/bmb/ldw052, 28069615

[31] Jonsdottir IH Rodjer L Hadzibajramovic E Borjesson M Ahlborg G Jr. A prospective study of leisure-time physical activity and mental health in Swedish health care workers and social insurance officers. Prev Med. (2010) 51:373–7. doi: 10.1016/j.ypmed.2010.07.019, 20691721

[32] Mendelsohn D Despot I Gooderham PA Singhal A Redekop GJ Toyota BD. Impact of work hours and sleep on well-being and burnout for physicians-in-training: the resident activity tracker evaluation study. Med Educ. (2019) 53:306–15. doi: 10.1111/medu.13757, 30485496

[33] Chin W Guo YL Hung YJ Yang CY Shiao JS. Short sleep duration is dose-dependently related to job strain and burnout in nurses: a cross sectional survey. Int J Nurs Stud. (2015) 52:297–306. doi: 10.1016/j.ijnurstu.2014.09.003, 25311378

[34] Chen Z Foo ZST Tang JY Sim MWC Lim BL Fong KY . Sleep quality and burnout: a Singapore study. Sleep Med. (2023) 102:205–12. doi: 10.1016/j.sleep.2022.12.026, 36706670

