# Supplementary material for: Mentally active versus passive sedentary behaviors and burnout among nurses of China: a cross-sectional study
Source: Front Public Health. 2026 Apr 21;14:1803165. doi: 10.3389/fpubh.2026.1803165 (PMC13139088; doi:10.3389/fpubh.2026.1803165)
Supplement: Supplementary file 1 [file Table_1.docx]

| Supplement table 1 Association between mentally passive SBs and burnout | | | | | | | | | |  |
| --- | --- | --- | --- | --- | --- | --- | --- | --- | --- | --- |
|  | Model 1 | | Model 2 | | | Model 3 | | |  |  |
|  | OR (95% CI) | P value | | OR (95% CI) | P value | | OR (95% CI) | P value | | |
| Napping |  |  | |  |  | |  |  | | |
| 0 | Ref |  | | Ref |  | | Ref |  | | |
| >0-≤1 | 0.73(0.53,1.02) | 0.066 | | 0.75(0.53,1.07) | 0.111 | | 0.83(0.57,1.19) | 0.304 | | |
| >1 | 1.43(0.69,2.84) | 0.313 | | 1.72(0.8,3.55) | 0.154 | | 1.98(0.9,4.21) | 0.081 | | |
| Watching TV |  |  | |  |  | |  |  | | |
| 0 | Ref |  | |  |  | | Ref |  | | |
| >0-≤1 | 0.76(0.54,1.06) | 0.109 | | 0.81(0.57,1.16) | 0.257 | | 0.82(0.63,1.17) | 0.278 | | |
| >1 | 0.65(0.41,1.02) | 0.068 | | 0.66(0.4,1.06) | 0.094 | | 0.74(0.44,1.21) | 0.243 | | |
| Browsing websites |  |  | |  |  | |  |  | | |
| 0 | Ref |  | | Ref |  | | Ref |  | | |
| >0-≤1 | 0.78(0.44,1.42) | 0.4 | | 0.75(0.4,1.43) | 0.372 | | 0.81(0.43,1.56) | 0.527 | | |
| >1 | 0.72(0.43,1.24) | 0.22 | | 0.76(0.43,1.21) | 0.354 | | 0.83(0.46,1.32) | 0.523 | | |
| Having meals |  |  | |  |  | |  |  | | |
| >0-≤1 | Ref |  | | Ref |  | | Ref |  | | |
| >1-≤2 | 0.98(0.69,1.38) | 0.907 | | 0.99(0.68,1.42) | 0.955 | | 0.99(0.68,1.43) | 0.959 | | |
| >2 | 1.18(0.68,1.98) | 0.536 | | 1.07(0.59,1.86) | 0.827 | | 1.1(0.6,1.94) | 0.762 | | |
| Model1 not adjusted;  Model 2 adjusted for age sex, BMI, education, monthly income, marital, children, smoke, drink, health status  Model 3: adjusted for all variables in Model 2 plus title, night shifts, sleep duration, and working years  *p < 0.05 | | | | | | | | | | |
|  | | | | | | | | | | |
| \| Supplement Fig1 \| \| \| --- \| --- \| \|  \|  \| \| Supplement Fig1 showcases a three-dimensional scatter diagram representing the relationship between emotional exhaustion (EE), depersonalization (DP), physical activity (PA), and different domains of sedentary behaviors (SBs). In the left graph, burnout is depicted along with the distribution of mentally active SBs. The x-axis represents EE, the y-axis represents DP, and the z-axis represents PA. It can be observed that as the mentally active SBs increase (indicated by lighter shades of green), they tend to converge towards the lower left corner. The right graph presents the association between mentally passive SBs and burnout. The color scheme utilized in this graph ranges from red (representing minimal mentally passive SBs) to blue (indicating more mentally passive SBs). Notably, the four colors are evenly distributed throughout the chart. \| \| | | | | | | | | | | |

| Supplement Fig 2 |
| --- |
| 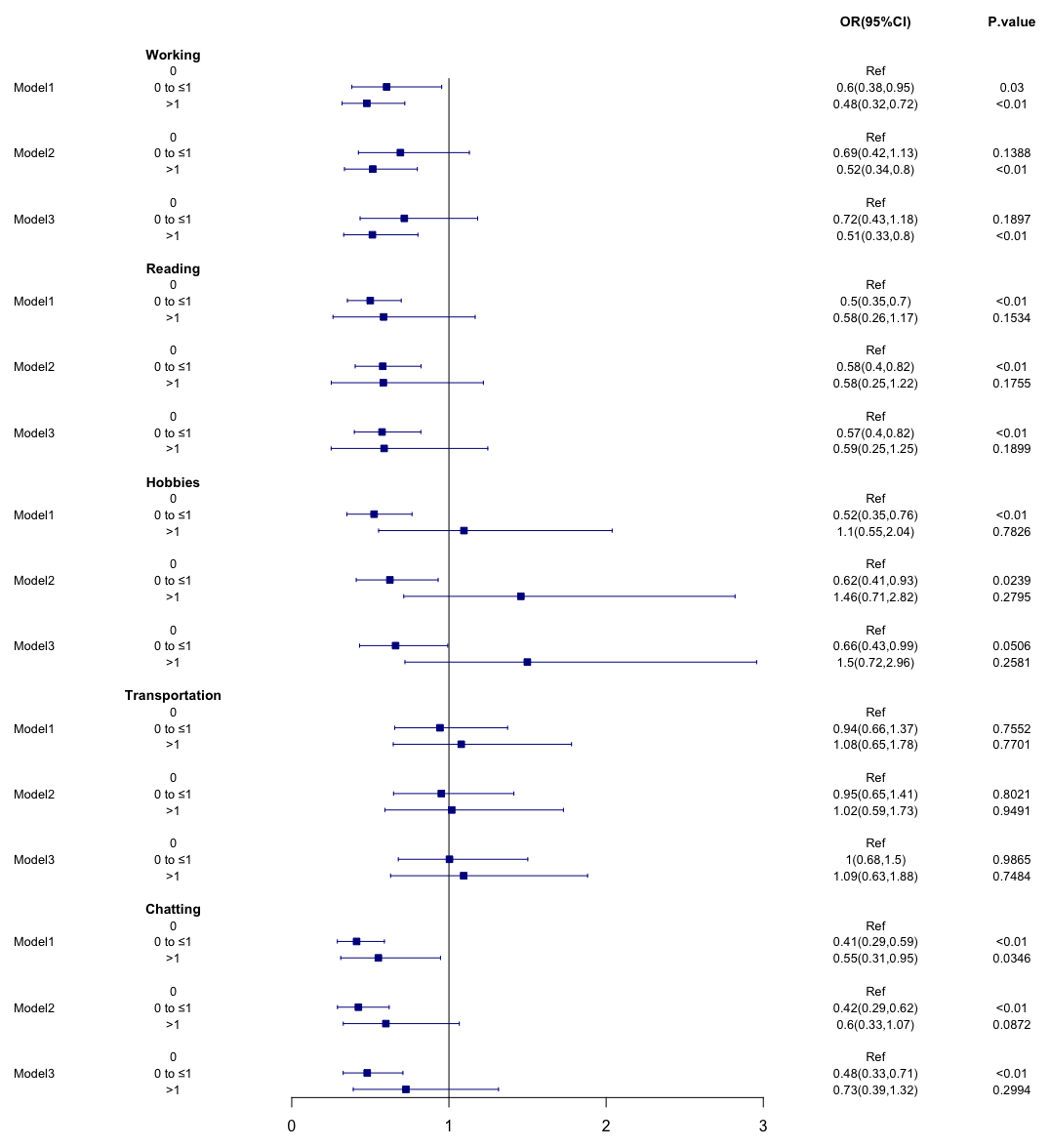 |
| Supplement Fig 2 presents a subgroup analysis focusing on mentally active SBs using different models. The specific mentally active SBs examined in the analysis are working, reading, hobbies, transportation, and chatting.  Model1 not adjusted; Model 2 adjusted for age sex, BMI, education, monthly income, marital, children, smoke, drink, healthy; Model 3: adjusted for all variables in Model 2 plus title, night shifts, sleep duration, and working years.  *p < 0.05 |
